# Role of 5-HT1A Receptor in the Anxiolytic-Relaxant Effects of Bergamot Essential Oil in Rodent

**DOI:** 10.3390/ijms21072597

**Published:** 2020-04-09

**Authors:** Laura Rombolà, Damiana Scuteri, Chizuko Watanabe, Shinobu Sakurada, Kengo Hamamura, Tsukasa Sakurada, Paolo Tonin, Maria Tiziana Corasaniti, Giacinto Bagetta, Luigi Antonio Morrone

**Affiliations:** 1 Preclinical and Translational Pharmacology, Department of Pharmacy, Health Science and Nutrition, University of Calabria, 87036 Rende, Italy; laura.rombola@unical.it (L.R.); damiana.scuteri@unical.it (D.S.); luigi.morrone@unical.it (L.A.M.); 2 Department of Physiology and Anatomy, Faculty of Pharmaceutical Sciences, Tohoku Medical and Pharmaceutical University, 4-4-1 Komatsushima, Aoba-ku, Sendai 981-8558, Japan; w-chizu@tohoku-mpu.ac.jp (C.W.); s-sakura@tohoku-mpu.ac.jp (S.S.); 3 Daiichi College of Pharmaceutical Sciences—First Department of Pharmacology Fukuoka, Fukuoka 815-8511, Japan; k-hamamura@daiichi-cps.ac.jp (K.H.); tsukasa@daiichi-cps.ac.jp (T.S.); 4 Regional Center for Serious Brain Injuries, S. Anna Institute, 88900 Crotone, Italy; patonin18@gmail.com; 5 Department of Health Sciences, University “Magna Graecia” of Catanzaro, 88100 Catanzaro, Italy; mtcorasa@unicz.it

**Keywords:** aromatherapy, bergamot essential oil, serotonin neurotransmission, anxiety and motor behavioral tests

## Abstract

The essential oil obtained by the fresh fruit of *Citrus bergamia* Risso et Poiteau is used worldwide in aromatherapy to reduce pain, facilitate sleep induction, and/or minimize the effects of stress-induced anxiety. Preclinical pharmacological data demonstrate that bergamot essential oil (BEO) modulates specific neurotransmissions and shows an anxiolytic-relaxant effect not superimposable to that of the benzodiazepine diazepam, suggesting that neurotransmissions, other than GABAergic, could be involved. Several studies on essential oils indicate a role for serotonergic (5-HT) neurotransmission in anxiety. Interestingly, among serotonergic receptors, the 5-HT1A subtype seems to play a key role in the control of anxiety. Here, we report that modulation of the 5-HT1A receptor by selective agonist ((±)8-OH-DPAT) or antagonist (WAY-100635) may influence some of the anxiolytic-relaxant effects of BEO in Open Field and Elevated Plus Maze tests.

## 1. Introduction

The essential oil obtained by cold press of the epicarp and, partly, the mesocarp of the fresh fruit of *Citrus bergamia*, Risso et Poiteau is used worldwide in aromatherapy to reduce pain, facilitate sleep induction, and/or minimize the effects of stress-induced anxiety [1,2,3,4]. These mood disorders and their comorbidity are two of the most debilitating psychiatric diseases that can compromise human well-being [5]. The global socioeconomic costs and suffering induced by mood disorders are of huge impact and concern in society [6]. In clinical practice, ongoing research for new drugs keeps a proper strategy towards the discovery of drugs with better pharmacological profile, particularly faster action and improved efficacy. Actually, the clinical successes of the application of many psychotropic drugs are characterized by non-response to treatment, non-adherence to prolonged treatment, and several side effects [7]. Approaches to natural products have been deeply studied [8,9,10,11], which could provide therapeutic options. Keeping in mind that mood disorders involve complex neural dysregulation, a clinically safe agent with several neural mechanisms could offer a better treatment. The use of bergamot essential oil (BEO) in aromatherapy is supported by pharmacological preclinical data obtained in different pain models [8,12,13,14,15,16] and in anxiety behavior tasks in rodents [17,18]. Particularly, BEO modulates EEG pattern [19] and neurotransmissions in freely moving rats [20]. Interestingly, the essential oil shows anxiolytic-relaxant effects in different, commonly used, behavioral tests not superimposable to those of benzodiazepine diazepam; particularly, no sedation is observed [17]. Moreover, in the Open Field test, the anxiolytic-relaxant effects of BEO are not affected by a pre-treatment with flumazenil, a benzodiazepine receptor antagonist [18]. The latter observation supports the hypothesis that neurotransmissions other than *γ*-aminobutyric acid (GABA)ergic could be involved. Serotonergic (5-HT) neurotransmission seems to play a crucial role in anxiety [21,22,23] and, incidentally, different pharmacological studies indicate that many essential oils used in aromatherapy modulate this neurotransmission [24,25,26,27,28,29]. Particularly, 5-HT1A receptor subtype, both as somatodendritic autoreceptor or as heteroreceptor on GABAergic and glutamatergic neurons [30,31], is involved in the control of anxiety influencing serotonergic neurotransmission in multiple brain regions. In the present study, we investigated the involvement of 5-HT1A receptors in the anxiolytic-relaxant activity of BEO in several behavioral tasks in rats. The animals were systemically pre-treated with a selective receptor antagonist, the compound *N*-{2-[4-(2-methoxyphenyl)-1-piperazinyl]ethyl}-N-(2-pyridinyl)cyclohexane-carboxamide trihydrochloride (WAY-100635) [32] or with the prototypical 5-HT1A receptor ligand 8-hydroxy-2-(di-n-propylamino) tetralin ((±)8-OH-DPAT) [33]. Elevated Plus Maze (EPM) and Open field (OF) tests were used to measure relaxant-anxiety effects, whereas Rotarod test was used to investigated motor impairment following drug treatments.

## 2. Results

### 2.1. Open Field Test

ANOVA analysis indicated that systemic administration of bergamot essential oil (BEO) (500 µL/kg) induced statistically significant differences between treatments for time spent in Immobility (F(6, 31) = 12,65; *p* < 0.0001), Grooming (F(6, 30) = 8,216; *p* < 0.0001), Center (F(6, 30) = 10,14; *p* < 0.0001), Crossing (F(6, 116) = 17,67; *p* < 0.0001), and Wallrearing (F(6, 116) = 19,95; *p* < 0.0001) frequencies.

**Figure 1:**
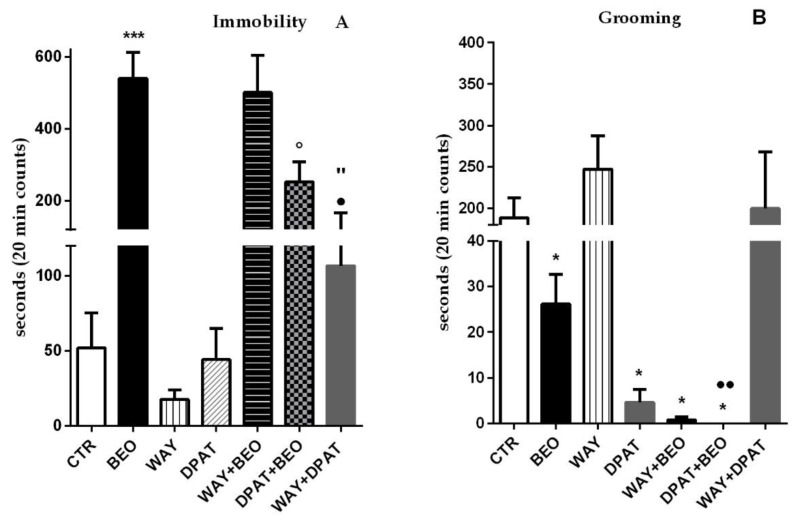
Immobility (**A**) and Grooming (**B**) in open field test in male Wistar rats after systemic (i.p.) administration of jojoba oil (CTR) (500 µL/kg), Bergamot Essential Oil (BEO) (500 µL/kg), WAY100635 (WAY) (1 mg/kg), 8-OH-DPAT (DPAT) (1 mg/kg), WAY + BEO (1 mg/kg + 500 µL/kg), DPAT + BEO (1 mg/kg + 500 µL/kg), and WAY + DPAT (1 mg/kg + 1 mg/kg). Data are expressed as mean ± SEM (*n* = 5–6 per group). * *p* < 0.05 and *** *p* < 0.001 vs. control group; ° *p* < 0.05 vs. BEO group; • *p* < 0.05 and •• *p* < 0.01 vs. DPAT group; “ *p* < 0.05 vs. WAY group. Statistical analysis was performed by one- or two-way ANOVA followed by individual comparisons with Tukey Multiple Comparison’s test.

Particularly, the animals treated with BEO showed a statistically significant increase in time spent not making movement with the head, body, paws, and tail (immobility) versus control group (Figure 1A).

**Figure 2:**
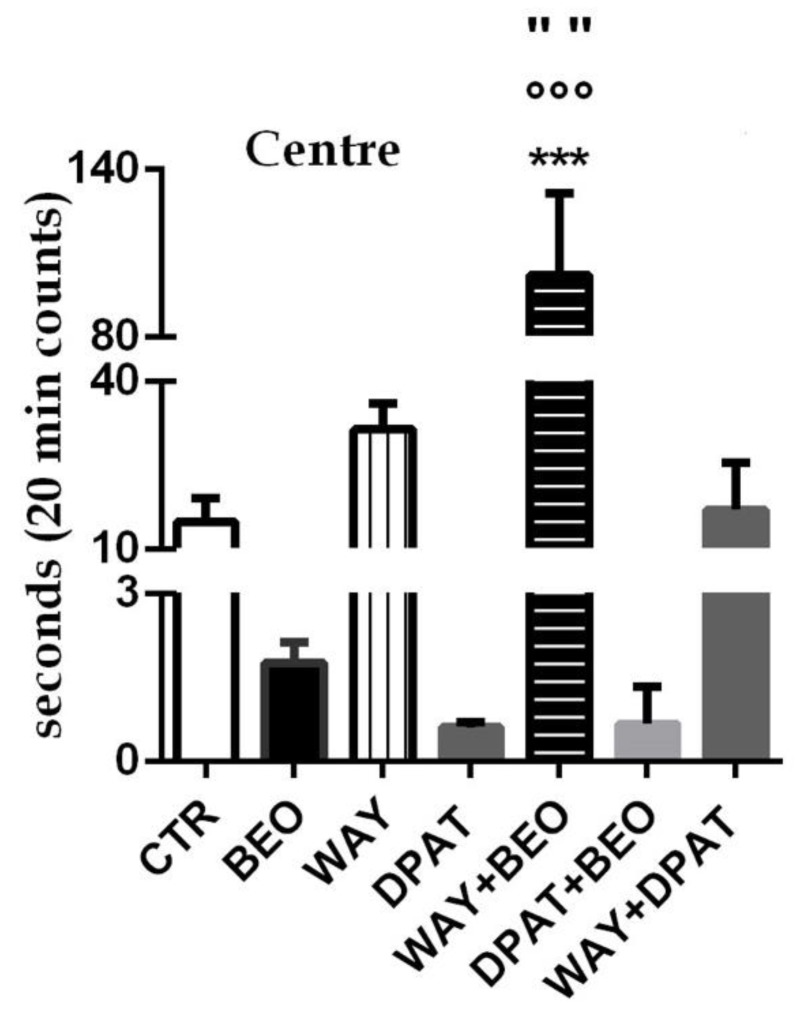
Time spent in the Center in open field test in male Wistar rats after systemic (i.p.) administration of jojoba oil (CTR) (500 µL/kg), Bergamot Essential Oil (BEO) (500 µL/kg), WAY100635 (WAY) (1 mg/kg), 8-OH-DPAT (DPAT) (1 mg/kg), WAY + BEO (1 mg/kg + 500 µL/kg), DPAT + BEO (1 mg/kg + 500 µL/kg), and WAY + DPAT (1 mg/kg + 1 mg/kg). Data are expressed as mean ± SEM (n = 5–6 per group). *** *p* < 0.001 vs. control group; °°° *p* < 0.001 vs. BEO group; “” *p* < 0.001 vs. WAY group. Statistical analysis was performed by one-way ANOVA followed by individual comparisons with Tukey Multiple Comparison’s test.

Conversely, the administration of (±)8-OH-DPAT or WAY-100635 did not induce any statistically significant change in immobility time when compared to control group (Figure 1A). Pre-treatment, 15 min before the essential oil, with the 5-HT1A antagonist did not affect BEO’s effect on immobility time, whereas the administration of serotonergic agonist reduced the behavior induced by BEO in a statistically significant way (Figure 1A). Interestingly, the administration of the 5-HT1A receptor antagonist, 15 min before the agonist, induced a significant increase in immobility time compared to both agonist and antagonist groups. Moreover, the animals treated with BEO spent less time performing grooming compared to control group (Figure 1B). Administration of WAY-100635 did not affect this behavior respect to control group, while the agonist (±)8-OH-DPAT showed a statistically significant decrease of it (Figure 1B). Interestingly, the pre-treatment with both the 5-HT1A agonist and antagonist markedly reduced in a statistically significant way the effect of the essential oil on grooming compared to control and BEO groups (Figure 1B). Moreover, the effect induced by (±)8-OH-DPAT was reversed by pre-treatment with WAY-100635 (Figure 1B). Analysis of the results also indicated that the animals treated with BEO or (±)8-OH-DPAT spent less time in the center of the arena when compared to control group (Figure 2).

Conversely, the administration of WAY-100635 showed a trend toward an increase of this behavior that did not reach statistical significance when compared to control group (Figure 2). Interestingly, the pre-treatment with WAY-100635 counteracted the effect of BEO and significantly increased the time rats spent in the center of the arena. Likewise, the effect induced by (±)8-OH-DPAT was reversed by pre-treatment with WAY-100635 but no statistically significant increase in the time spent in the center of the arena was observed when compared to control group. The pre-treatment with the 5-HT1A agonist did not modify the effect of BEO (Figure 2).

**Figure 3:**
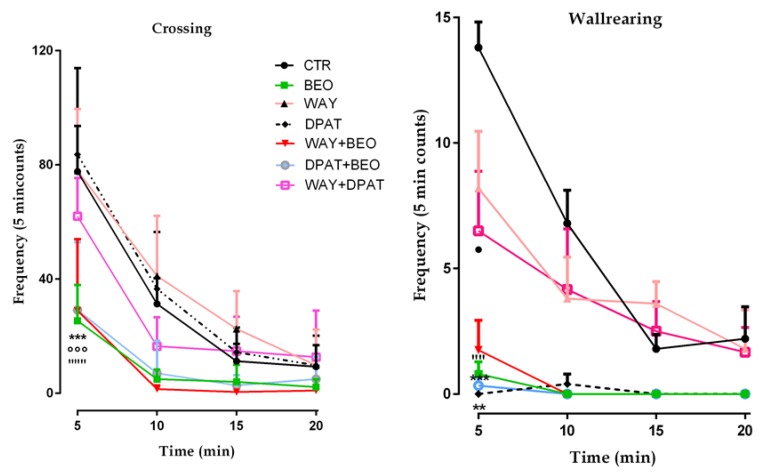
Crossing and Wallrearing frequencies in open field test in male Wistar rats after systemic (i.p.) administration of jojoba oil (CTR) (500 µL/kg), Bergamot Essential Oil (BEO) (500 µL/kg), WAY100635 (WAY) (1 mg/kg), 8-OH-DPAT (DPAT) (1 mg/kg), WAY + BEO (1 mg/kg + 500 µL/kg), DPAT + BEO (1 mg/kg + 500 µL/kg), and WAY + DPAT (1 mg/kg + 1 mg/kg). Data are expressed as mean ± SEM (*n* = 5–6 per group). *** *p* < 0.001 and ** *p* < 0.01 vs. control group; °°° *p* < 0.001 vs. BEO group; • *p* < 0.05 vs. DPTA group; “” *p* < 0.01 and “”” *p* < 0.001 vs. WAY group. Statistical analysis was performed by one-way ANOVA followed by individual comparisons with Tukey Multiple Comparison’s test.

Analysis of the data also showed that there was a statistically significant difference for crossing and wallrearing frequencies in the animals treated with BEO when compared to control group (Figure 3) and this effect was not modified when the animals were pre-treated with either the agonist or the antagonist of 5-HT1A receptor (Figure 3).

Moreover, the animals treated with WAY-100635 or (±)8-OH-DPAT did not modify this behavior when compared to control group, whereas a statistically significant decrease of wallrearing was observed in the animals treated with (±)8-OH-DPTA (Figure 3).

### 2.2. Elevated Plus Maze Test

Statistical analysis performed by one-way ANOVA indicated statistically significant differences after drug treatments considering the percentage of time spent in open arms (F(6, 38) = 5,782; *p* = 0.0002), time spent in closed arms (F(6, 38) = 4,946; *p* = 0.0008), and number of entries in closed arms (F(6, 38) = 2,888; *p* = 0.0203). However, no statistically significant differences were calculated after drug treatments considering the number of entries in open arms (F(6, 38) = 1,494; *p* = 0.2066).

**Figure 4:**
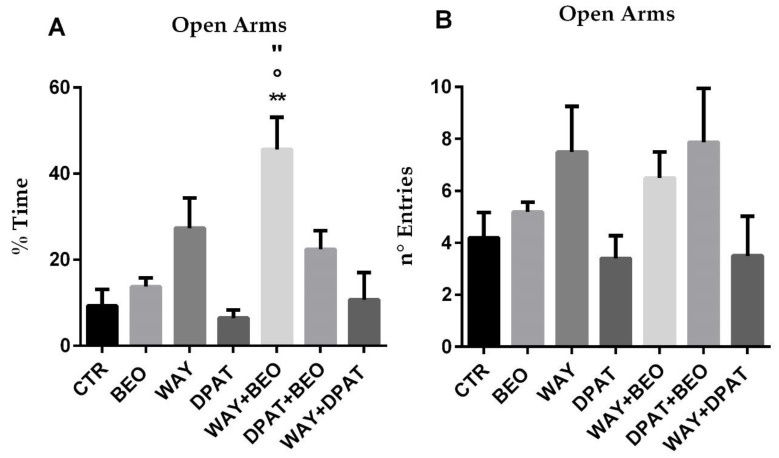
% of Time (**A**) and n° of Entries (**B**) in open arms in the elevated plus-maze test in male Wistar rats after systemic (i.p.) administration of jojoba oil (CTR) (500 µL/kg), Bergamot Essential Oil (BEO) (500 µL/kg), WAY100635 (WAY) (1 mg/kg), 8-OH-DPAT (DPAT) (1 mg/kg), WAY + BEO (1 mg/kg + 500 µL/kg), DPAT + BEO (1 mg/kg + 500 µL/kg), and WAY + DPAT (1 mg/kg + 1 mg/kg). Data are expressed as mean ± SEM (*n* = 5–8 per group). ** *p* < 0.01 vs. control group; ° *p* < 0.05 vs. BEO group; “ *p* < 0.05 vs. WAY group. Statistical analysis was performed by one-way ANOVA followed by individual comparisons with Tukey Multiple Comparison’s test.

Particularly, the administration of BEO and WAY-100635 showed a trend toward an increase percentage of time spent in open arms compared to control group without reaching statistical significance (Figure 4A). Conversely, the administration of (±)8-OH-DPAT showed a trend toward a decrease percentage of time spent in open arms compared to control group without reaching statistical significance (Figure 4A). Interestingly, pre-treatment with both the 5-HT1A agonist and the antagonist, 15 min before BEO, induced an increase percentage of time spent in open arms but statistical significance was only achieved with WAY-100635 (Figure 4A). Pre-treatment with WAY-100635 did not modify the effect of (±)8-OH-DPAT (Figure 4A). Although the number of entries in open arms showed a similar trend to the percentage of time, no statistically significant differences were calculated (Figure 4B).

**Figure 5:**
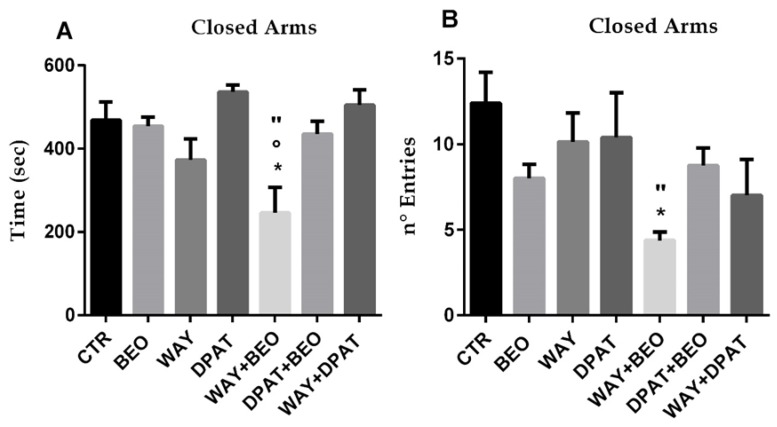
Time spent (**A**) and Number of Entries (**B**) in closed Arms in the elevated plus-maze test in male Wistar rats after systemic (i.p.) administration of jojoba oil (CTR) (500 µL/kg), Bergamot Essential Oil (BEO) (500 µL/kg), WAY100635 (WAY) (1 mg/kg), 8-OH-DPAT (DPAT) (1 mg/kg), WAY + BEO (1 mg/kg + 500 µL/kg), DPAT + BEO (1 mg/kg + 500 µL/kg), and WAY + DPAT (1 mg/kg + 1 mg/kg). Data are expressed as mean ± SEM (*n* = 5–8 per group). * *p* < 0.05 vs. control group; ° *p* < 0.05 vs. BEO group; “ *p* < 0.05 vs. WAY group. Statistical analysis was performed by one-way ANOVA followed by individual comparisons with Tukey Multiple Comparison’s test.

The administration of BEO did not affect the time spent in closed arms (Figure 5A).

However, it induced a decrease in number of entries (Figure 5B). Pre-treatment with WAY-100635 induced a statistically significant decrease in time spent (Figure 5A) and in number of entries (Figure 5B) in closed arms in animals treated with BEO compared to control or WAY-100635 groups.

### 2.3. Rotarod Test

**Figure 6:**
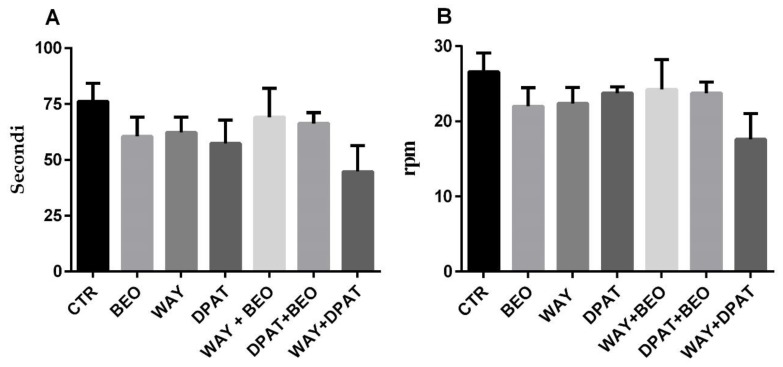
Time (**A**) and rpm (**B**) in rotarod in male Wistar rats after systemic (i.p.) administration of jojoba oil (CTR) (500 µL/kg), Bergamot Essential Oil (BEO) (500 µL/kg), WAY100635 (WAY) (1 mg/kg), 8-OH-DPAT (DPAT) (1 mg/kg), WAY + BEO (1 mg/kg + 500 µL/kg), DPAT + BEO (1 mg/kg + 500 µL/kg), and WAY + DPAT (1 mg/kg + 1 mg/kg). Data are expressed as mean ± SEM (*n* = 5–6 per group). Statistical analysis was performed by one-way ANOVA followed by individual comparisons with Tukey Multiple Comparison’s test.

The treatment with all the drugs tested did not induce differences of time and revolutions per minute (rpm) values measured when animal falls off rod (Figure 6A,B). Statistical analysis performed by one-way ANOVA indicated no statistically significant differences after drug treatments considering the time (F(6, 36) = 0.9127; *p* = 0.4970) and rpm (F(6, 35) = 0.821; *p* = 0.5677).

### 2.4. Statistics

Statistical analyses were carried out using Graph Pad® 6.0 for Windows. Results (mean ± SEM, (*n* = 5–8 per group) were tested for normality by the selection of parametric and non-parametric tests. Behavioral data were analyzed by ordinary one or two (for treatments only) way analysis of variance (ANOVA) followed by Tukey Multiple Comparison’s test. Differences were considered significant only when *p*-value is *p* < 0.05.

## 3. Discussion

The results of this study confirm that BEO is endowed with a relaxant-anxiolytic effect in animal behavioral tasks. In fact, it increased immobility time and reduced grooming in OF task and increased the percentage of time spent in open arms in EPM task when compared to control group [17]. Previously, we demonstrated that these effects of the essential oil are not superimposable to those of benzodiazepine diazepam and no sedation is observed [17]. Furthermore, pre-treatment with the benzodiazepine receptor antagonist flumazenil does not counteract these effects of BEO [18], suggesting that neurotransmissions, other than GABAergic, are involved. Particularly, the serotonin system plays an important role in the neural processing of anxiety [34,35,36] and, among the central serotonin receptors (5-HT1-7), the 5-HT1A receptor subtype seems to play a key role in the control of anxiety by influencing serotonergic neurotransmission in multiple brain regions [30,35,36,37,41]. The latter include the raphe nuclei, where 5-HT1A is expressed as somatodendritic autoreceptor [38], and in other brain regions such as hippocampus and cortex, where this subtype is also expressed as a heteroreceptor on glutamatergic and GABAergic neurons [30,39]. 5-HT ligands that stimulate postsynaptic 5-HT1A receptors in terminal areas of serotonergic projections show an anxiogenic profile (e.g., [30]). On the other hand, compounds that stimulate inhibitory somatodendritic 5-HT1A autoreceptors in the raphe nuclei, decrease the firing frequency of 5-HT neurons and, hence, reduce 5-HT release, showing anxiolytic effects [30,40]. The compound WAY-100635 has a potent and selective antagonistic action at both pre- and postsynaptic 5-HT1A receptor sites [32,41,42]. Hence, although WAY-100635 can inhibit hippocampal cell firing [42] and decrease serotonin concentration in the hippocampus and nucleus accumbens [43,44], it can also prevent 5-HT1A receptor-mediated auto-inhibition of the firing frequency of 5-HT neurons in the raphe nuclei [32,42,45,46]. Interestingly, literature data indicate that WAY-100635 induces anxiolytic effects in animal behavioral tasks [47,48,49,50,51,52,53] and our results in OF and EPM are in agreement with these data. Several studies using 5-HT1A receptor agonists, however, have yielded highly variable results in different anxiety tests, particularly for compounds administered systemically (for a review, see [54]). (±)8-OH-DPAT is the standard selective 5-HT1A agonist [33] and its systemic injection shows anxiogenic or no effects [33,47,48,49,55]. Our results in OF and EPM tasks are in agreement with these data and support them. 

To gain more insight regarding the mechanisms involved in anxiolytic-relaxant effects of BEO, we investigated the involvement of 5-HT1A receptors in several animal behavioral tasks. 

Altogether, our data seem to suggest that the modulation of 5-HT1A receptors may interfere with some of the anxiolytic-relaxing effects of BEO but that the effects of the essential oil do not occur through the activation of these receptors. The analysis of individual behaviors seems to support this hypothesis. For example, the increase of immobility time elicited by the essential oil in OF task is not counteracted by WAY-100635, suggesting that 5-HT1A receptors are not involved. On the other hand, the ability of (±)8-OH-DPAT to reduce the freezing effect of the essential oil could be due to the activation of pre-synaptic 5-HT1A receptors resulting in modulation of release of other neurotransmitters such as GABA and glutamate [39]. Interestingly, glutamatergic transmission appears to play a key role in immobility behavior [56,57,58] and neurochemical data demonstrate that BEO is able to increase glutamatergic transmission in hippocampus of rat [20]. Therefore, it is likely that (±)8-OH-DPAT, acting on pre-synaptic 5-HT1A receptors, reduces glutamate release [59] and then the effect of BEO on immobility time. Conversely, the effect obtained by the interaction between antagonist and agonist is not easily understood, suggesting that complex self-adjustments in the serotonergic system may be involved. The relaxing action of BEO was also reflected on grooming behavior that was significantly reduced compared to control group. Similarly, a marked reduction in grooming was achieved with (±)8-OH-DPAT. This result suggests that BEO may be able to reduce grooming by activating 5-HT1A receptors; however, while the effect of (±)8-OH-DPAT was reversed by WAY-100635, the effect of the essential oil on grooming was not counteracted by the 5-HT1A receptors antagonist and, indeed, appeared to be enhanced. The mechanism underlying the enhancement of the effect of BEO by WAY-100635 is not clear, whereas it is likely that the enhancement of the same effect by (±)8-OH-DPAT could derive from the synergy of different neurotransmitter systems. A condition similar to that observed for grooming was also present in the analysis of the time spent in the center of arena by animals. In fact, rats treated both with BEO and (±)8-OH-DPAT spent less time in the center when compared to control group. Interestingly, the pre-treatment with WAY-100635 counteracted the effect of both BEO and (±)8-OH-DPAT but significantly increased the time spent in the center of arena in animals treated with BEO given alone. We speculate that the observed effect may stem from the synergistic action among different neurotransmissions involved rather than to the displacement from the receptor sites by WAY-100635. WAY-100635 would block post-synaptic 5-HT1A receptor and the increased release of glutamate by BEO could lead to the activation of metabotropic glutamate receptors 8 (mGluR8) involved in anxiety disorders [60,61,62]. The modulation of 5-HT1A receptors seems also not to interfere with BEO effects on crossing and wallrearing frequencies.

The lack of a direct involvement of the 5-HT1A receptor in the effects of BEO was also observed in elevated plus maze (EPM) task. The anxiolytic effect of BEO was measured by an increase percentage of time spent in the open arms that, however, did not reach a statistically significant difference when compared to control group. Interestingly, pre-treatment with WAY-100635 enhanced the anxiolytic effect of BEO. In fact, the percentage of time spent in open arms was increased and reached a statistically significant difference when compared to control and BEO groups. Similar to what it was observed in center behavior, it is likely that these results could be correlated to a synergistic effect between the blockade of post-synaptic 5-HT1A receptors by WAY-100635 and the agonistic action of glutamate released by BEO on mGluR8 [60,61,62]. The latter hypothesis could explain the marked decrease by WAY-100635 in time spent and in number of entries in closed arms in animals treated with BEO. Conversely, the increase of the percentage of time in open arms observed in the animals pre-treated with (±)8-OH-DPAT and then with BEO is not clear and easily understandable. Finally, no motor impairment was observed in the rotarod test after drug treatments and the modulation of 5-HT1A receptors did not modify the effect of BEO.

## 4. Materials and Methods 

### 4.1. Animals

The procedures were performed in accordance with the conventional guidelines for animal experimentation (Italian D.L. No. 26/2014 and subsequent variations) and the recommendations of the European Economic Community (2010/63/UE). The experimental protocols were authorized by the Ministry of Health (Rome, Italy; Authorization Number 305/2019-PR date of approval: 10 April 2019). Male Wistar rats (Charles River, Lecco, Italy) weighing 200–275 g were used, being the most used in this type of experiments. Animals were individually housed in 36 cm × 18.5 cm × 24 cm (shoebox) clear polyethylene cages provided with a wood shavings bedding, with food (VRF1 feed) and tap water available ad libitum. The colony room was held under a 12 h light and 12 h dark cycle (light 07:00–19:00 h at 79.9 lux or 0.12 W/m^2^) at 20–22 °C. Rats were allowed a minimum of seven days to recover from the stress of shipping before any procedures. Animals were provided with free access to the standard rodent diet and purified drinking water till the completion of the study. The cages holding two rats were changed once weekly and animals were never tested on the two days following the changing. Each animal was tested only once in the tests.

### 4.2. Experimental Analysis 

Behavioral testing was carried out as reported by Rombolà and colleagues [17,18]. Briefly, rats were weighed and individually placed in a cleaned opaque cage with highest walls to prevent it from escaping, transported to the testing room and leaved for one hour to the new environment.

Studies conducted with similar methods and with similar endpoints [24,25,64] indicate a number of animals between 5 and 10 to evaluate the involvement of specific neurotransmissions on the anti-stress and anxiolytic effects of various essential oils. An a priori analysis of the power of the study conducted with the G * Power program, using the means and the standard error (transformed into standard deviation), allowed to calculate an average effect size equal to 1.76 and then to determine the minimum sample size needed to obtain a statistical power of 0.80 and an alpha of 0.05. The animals were randomly assigned to seven experimental groups (*n* = 5–8 per group) as follows: saline + jojoba oil (CTR), saline + BEO (BEO), WAY-100635 + jojoba oil (WAY), WAY-100635 + BEO (WAY + BEO), (±)8-OH-DPAT + jojba oil (DPAT), (±)8-OH-DPAT + BEO (DPAT + BEO), and WAY-100635 + (±)8-OH-DPAT (WAY + DPAT). Between two consecutive intraperitoneal (i.p.) injections, there was an adjustment interval of 15 min. Thirty minutes after the second injection, the behavioral effects of the different treatments were evaluated by using EPM, OF, and Rotarod tasks. The schedule experimental protocol is as follows:


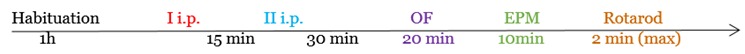


Bergamot essential oil was kindly provided by “Capua Company1880 S.r.l.”, Campo Calabro, Reggio Calabria (Italy); the certificate of analysis indicates that the essential oil of bergamot contains d-limonene, 39.60%; linalyl acetate, 31.09%; linalool, 9.55%. The dose of BEO of 500 µL/kg was used according to previous studies [17,18,20]. (±)8-OH-DPAT and WAY-100635 were purchased by Tocris Cookson, Inc. (Bristol, UK) and used at 1 mg/kg dissolved in saline solution. This dose is used to study the role of 5-HT1A receptor in anxiety and the interference with the effects of essential oils [25,63,64].

The behavior of the animal during EPM, OF, and rotarod tasks were registered consecutively with a closed-circuit camera between 09.00 and 14.00, during the light phase of the circadian rhythms of the animal. Behavioral sessions were videotaped for further analysis by a trained observer who remained blind to drug treatments. At the end of the experiment, the animal was euthanized by an overdose of isoflurane. The experimental room was ventilated and all behavioral apparatus were washed with water and cleaned up with 70% ethanol after daily sessions to avoid any remaining odor traces of the essential oil and excrements. 

### 4.3. Open Field Test

The apparatus consisted of a dark, plastic circular arena (75-cm diameter) placed in a dim lighting (~40 lux) and sound proof chamber, as previously described by Walsh and Cummins [65]. The base area was divided into eight equal sectors. A single rat was placed in the center of the arena and during 20 min different behaviors were registered. To measure general locomotor activity, the following behavioral parameters (expressed as frequency on 5 min counts) were scored: number of square limit crossings with both forepaws and wallrearing (touching the walls of the apparatus with the forelimbs standing on the hind-limbs). To assess anxiety-related behavior, additional parameters were measured: (a) time spent in the center of arena; and (b) time spent performing general grooming activity consisting of face grooming (strokes along the snout), head washing (semi-circular movements over the top of the head and behind the ears), and body grooming (body fur licking). Besides, time spent by the rat not making movement with the head, body, paws, and tail (immobility) was measured. The scoring was performed using a video-tracking motion analysis system (Labehaviour).

### 4.4. Elevated Plus Maze Test

The EPM testing procedure is an “unconditional” anxiety-like test based on rodents “natural aversion” to open space and heights and it was based on a method described by Pellow et al. (1985) [66]. The EPM apparatus was made of durable, high density, black plexiglass, elevated to a height of 50 cm, consisted of two open arms (50 × 10 cm^2^) and two closed arms (50 × 10 cm^2^) and 40 cm high walls, arranged so that the two arms of each type were opposite each other. Each animal was gently placed in the center of the EPM, facing one of the open arms and, during the 10 min test period, the time spent in the open and closed arms and the number of entries in either type of the arms were recorded. An effective entry was characterized as all four paws of the animal having crossed the line between an arm and central area. During a 10 min observation period, the time (seconds) spent in open and closed arms and the number of open and closed arm entries were calculated. The percent open arm time, an inverse measure of anxiety-like behavior, was calculated as (time in open arms/total time in arms) × 100.

### 4.5. Rotarod Test

The rotarod test was performed according to the method described by Jones and Roberts [67], to measure motor coordinating activity of the rats by their performance on an accelerating rotarod (47750 Rota-Rod NG, Ugo Basile, Italy). The apparatus consisted of four 6-cm diameter cylinders, which were suitably machined to provide grip. Five 49-cm diameter dividers made four lanes, each 8.7 cm wide with a height to fall of 30 cm. To avoid to take into account the inability to stay on the rotating rod and to habituate the animal to the apparatus, rats were pre-selected one day before the test on the rotating rod. The rat was held by the tail and placed on the rotating rod at 5 rpm, facing away from the direction of rotation so it had to walk forward to stay upright. If the animal fell before 5 s, it was due to poor placing by the experimenter so the animal was repositioned. After 60 s on the rod, animal was returned to home cage. The procedure was repeated for a total of three trials separated by 10-min inter-trial intervals. 

The day of the test, the apparatus was set to accelerate from 4 to 40 rpm in 120 s, and the animal was placed on the rod initially rotating at 4 rpm. The trial began when acceleration was started and ended when the animal fell off the rod. The timer was stopped and time and rpm values were registered. The animal that clung to the rod and completed full passive rotation was given up on.

## 5. Conclusions

Our results seem to suggest that the anxiolytic-relaxant effects of BEO observed in OF and EPM tasks do not arise from a direct involvement of 5-HT1A receptors, although their modulation seems to be able to indirectly interfere with BEO activity. For some of these effects of the essential oil, we speculate a role for glutamatergic system that needs to be investigated thoroughly [68]. Altogether, these data confirm that anxiolytic-relaxant effects of BEO underlie the involvement of multiple, complex, mechanisms and contribute to the characterization of the neurobiological profile of BEO for its rational use in aromatherapy. In this context, the enhancement of anxiolytic effects observed by synergistic action between BEO and the 5-HT1A antagonist in OF and EPM tasks are of particular interest and deserve further investigation.

## Abbreviations

(±)8-OH-DPAT	8-hydroxy-2-(di-n-propylamino) tetralin
5-HT	Serotonin
BEO	Bergamot essential oil
CTR	Control
EPM	Elevated Plus Maze
mGluR8	Metabotropic glutamate receptors8
OF	Open Field
WAY-100635	N-{2-[4-(2-methoxyphenyl)-1-piperazinyl]ethyl}-N-(2-pyridinyl)cyclohexanecarboxamide trihydrochloride
